# The Royal College of Surgeons

**Published:** 1899-07-15

**Authors:** 


### THE ROYAL COLLEGE OF SURGEONS.

The perennial quarrel between a certain number of the members of the Royal College of Surgeons and the fellows and council thereof goes gently on, and showed its head once more at the meeting recently held to consider the application for a new charter. The members resolve all sorts of things, especially that they should have some representation on the council. The fellows meanwhile block the way, or at least Mr. Pickering Pick says they do, and behind them sit serene, impartial, and undisturbed, the members of the council. That is one view of the situation. The other is that held by Mr. George Brown, who apparently looks upon these gentlemen as so many Machiavels, pulling wires to the destruction of the members' hopes, for he held that the fellows would vote in favour of the members' demand if they were not instructed by the council how to vote. In the end the meeting resolved and voted strongly against the council.

